# Combination of Serological, Antigen Detection, and DNA Data for *Plasmodium falciparum* Provides Robust Geospatial Estimates for Malaria Transmission in Haiti

**DOI:** 10.1038/s41598-020-65419-w

**Published:** 2020-05-21

**Authors:** Adan Oviedo, Alaine Knipes, Caitlin Worrell, LeAnne M. Fox, Luccene Desir, Carl Fayette, Alain Javel, Franck Monestime, Kimberly Mace, Michelle A. Chang, Venkatachalam Udhayakumar, Jean F. Lemoine, Kimberly Won, Patrick J. Lammie, Eric Rogier

**Affiliations:** 1 Rollins School of Public Health, Emory University, Atlanta, GA 30322 US; 2 Division of Parasitic Diseases and Malaria, Centers for Disease Control and Prevention, Atlanta, GA 30329 US; 3 The Carter Center, Atlanta, GA 30307 US; 4 IMA World Health, Port-au-Prince, Haiti; 5 Programme National de Contrôle de la Malaria, Ministère de la Santé Publique et de la Population (MSPP), Port-au-Prince, Haiti; 6 Task Force for Global Health, Decatur, GA 30030 US

**Keywords:** Diagnostic markers, Predictive markers, Biomarkers, Malaria, Immunological techniques

## Abstract

Microscopy is the gold standard for malaria epidemiology, but laboratory and point-of-care (POC) tests detecting parasite antigen, DNA, and human antibodies against malaria have expanded this capacity. The island nation of Haiti is endemic for *Plasmodium falciparum* (*Pf*) malaria, though at a low national prevalence and heterogenous geospatial distribution. In 2015 and 2016, serosurveys were performed of children (ages 6–7 years) sampled in schools in Saut d’Eau commune (n = 1,230) and Grand Anse department (n = 1,664) of Haiti. Children received malaria antigen rapid diagnostic test and provided a filter paper blood sample for further laboratory analysis of the *Pf* histidine-rich protein 2 (HRP2) antigen, *Pf* DNA, and anti-*Pf* IgG antibodies. Prevalence of *Pf* infection ranged from 0.0–16.7% in 53 Saut d’Eau schools, and 0.0–23.8% in 56 Grand Anse schools. Anti-*Pf* antibody carriage exceeded 80% of students in some schools from both study sites. Geospatial prediction ellipses were created to indicate clustering of positive tests within the survey areas and overlay of all prediction ellipses for the different types of data revealed regions with high likelihood of active and ongoing *Pf* malaria transmission. The geospatial utilization of different types of *Pf* data can provide high confidence for spatial epidemiology of the parasite.

## Introduction

Malaria is caused by parasites within the genus *Plasmodium* and is found in most tropical regions throughout the world^1^. Elimination efforts in the 1900s and 2000s have reduced the number of countries with endemic malaria to 87, and 26 nations (as of the year 2018) are near elimination as defined by fewer than 100 indigenous cases per year^2^. The reduction in malaria transmission in a region is an accomplishment to be greatly celebrated, but also conveys practical challenges as a nation tries to move from a low incidence of malaria to zero malaria. With reduction of disease prevalence, funds for malaria control/elimination are often redirected to other public health efforts^2–4^. As the incidence of malaria infections decrease in a country, the capacity of health care facilities for accurate and sensitive malaria diagnosis and the availability of appropriate diagnostic tools also declines^5,6^. The concept of asymptomatic, low parasite density carriage in a population becomes much more important when planning to eliminate the entire parasite reservoir^7,8^, but current routine diagnostic tests are not ideal for identification of asymptomatic infections.

To estimate malaria prevalence or transmission in an area, multiple tests are available to identify active infections, recent infections, or past exposure for individuals in the population. Historically, light microscopy for examination of blood films has been the gold standard for identification of malaria parasites with visual verification of the organism, and has been in use for over 100 years^6,9^. More recently, field-deployable antigen detection tests have allowed for the widespread use of a malaria diagnostic which requires minimal user training and identifies infections with sensitivity matching or exceeding microscopy^10^. In the laboratory, detection of malaria parasite DNA provides a highly-sensitive measure of *Plasmodium* in the blood, but these assays are expensive and laborious to use on a routine basis in especially resource limited settings due to multiple processing steps and costly reagents^11^. Laboratory assays have recently been developed for the ultrasensitive detection of malaria antigens^12,13^. The presence of malaria antigens such as pan-*Plasmodium* aldolase and lactate dehydrogenase (LDH) indicate either an active infection or parasite clearance within the past 1–2 weeks^14^, whereas the *P. falciparum* histidine-rich protein 2 (HRP2) antigen is found in blood during active infection, but also for several months following parasite clearance^15^. Antibodies against malaria parasite antigens are a very different type of indicator for malaria exposure in that they are produced by the host adaptive immune system, and not directly by the parasite. Antibody markers can serve as indicators for historical malaria exposure, and numerous malaria antigens have now been identified that are known to induce both short- and long-lived IgG responses (months in duration to years in duration)^16–18^.

In this study, we measure and compare multiple indicators of malaria infection or exposure from school-based surveys in two locations in Haiti conducted in conjunction with lymphatic filariasis transmission assessment surveys. Malaria in Haiti is predominantly caused by *P. falciparum* (*Pf*), has highly heterogeneous transmission patterns, and is at a nationwide infection prevalence of under 1%^19^. Results for antigen-based field diagnostics, malaria DNA assays, laboratory antigen tests, and IgG antibody detection assays were all obtained from blood samples provided by children (ages 6–7 years) participating in the study and geospatial clustering of positives was investigated. As Haiti and other countries near malaria elimination, identifying the most practical and robust tools for malaria surveillance will aid in finding the last reservoirs of *Pf* parasites in the population.

## Results

In total, 2,894 children were enrolled; 1,230 children from 53 Saut d’Eau schools and 1,664 children from 56 Grand Anse schools (Table 1). The median number of children enrolled per school in each study site was the same (n = 27) with approximately a 1:1 ratio of males to females (48.7% versus 50.1%, respectively; that information not captured for 1.2%). Slightly higher numbers of 7-year olds were enrolled versus 6-year olds (58.2% versus 40.6%, respectively; not captured for 1.2%). For both study sites, the proportion of children with as malaria positive (determined by RDT, PET-PCR, or HRP2 lab assay) varied dependent on the test administered and was found to be overall quite low (≤ 2.1%)(Table 1). In contrast, the proportion of children seropositive for anti-malarial IgG antibodies was much higher, likely indicative of past *Pf* exposure for these children. In Saut d’Eau commune, 4.4% of children were positive for short-term IgG (likely acquired over the past year) against *Pf* whereas 33.7% were positive for long-term IgG (acquired at some point in life). In comparison to Saut d’Eau, children in Grand Anse demonstrated slightly lower short-term IgG carriage (3.1%) and significantly lower long-term IgG carriage (18.7%).

**Table 1:** Demographic characteristics of study populations and point estimates.

Characteristics	Saut d’Eau Commune	Grand Anse department
Number Enrolled	1,230	1,664
Female (%)	650 (52.8%)	800 (48.1%)
Male (%)	580 (47.2%)	829 (49.8%)
Not provided (%)	0 (0.0%)	35 (2.1%)
Age at enrollment (years)		
6 (%)	478 (38.8%)	697 (41.9%)
7 (%)	753 (61.2%)	932 (56.0%)
Not provided (%)	0 (0.0%)	35 (2.1%)
Number schools sampled	53	56
Median number students enrolled at schools	27	27
Range number students enrolled at schools	1–74	1–133
RDT positive (%, 95% CI)	7 (0.57%, 0.15–0.99)	16 (1.9%, 1.0–2.9)*
PET-PCR positive (%, 95% CI)	9 (0.73%, 0.26–1.2)	NA
HRP2 antigen positive (%, 95% CI)	26 (2.1%, 1.3–2.9)	24 (1.4%, 0.87–2.0)
Short-term IgG positive (%, 95% CI)	55 (4.4%, 3.3–5.6)	52 (3.1%, 2.3–3.9)
Long-term IgG positive (%, 95% CI)	416 (33.7%, 31.0–36.3)	311 (18.7%, 16.7–20.4)*

*, Overall test prevalence between study sites is significantly different (p < 0.05).

**Figure 1:**
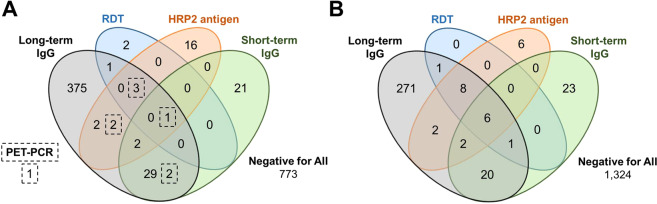
Concordance of Different Indicators of *Plasmodium falciparum* Infection or Exposure. Venn diagrams are shown for field and laboratory test results for 1,230 children from Saut d’Eau commune (**A**) and 1,664 children from Grand Anse department (**B**). Additional PET-PCR assay completed for all Saut d’Eau samples with positives in hashed boxes in their appropriate Venn diagram categories.

As all tests (with the exception of HRP2-based RDT and the HRP2 lab assay) measure unique markers of malaria infection or exposure, concordance among all tests was highly variable (Fig. 1). In Saut d’Eau, only one child was found to be positive for all five tests, whereas 773 children (62.8%) were negative for all. For each of the five tests, participants were found be positive for only that test, with long-term IgG having the highest number of these (375, 30.5% of Saut d’Eau children enrolled). In Grand Anse (where PET-PCR was not performed), 6 children (0.4%) were positive for all four tests and 1,324 (79.6%) negative for all. With the exception of RDT, the three other *Pf* tests had participants positive only for that test, with long-term IgG again having the highest number of these single-positives (271, 16.3% of all Grand Anse children enrolled). In Saut d’Eau, RDT and PET-PCR diagnostic test positivity were associated with significantly higher short- and long-term anti-*Pf* IgG levels (Supplemental Fig. 1A,B, respectively). In Grand Anse, RDT and lab HRP2 assay positivity were both associated with increased short- and long-term anti-*Pf* IgG levels (Supplemental Fig. 1C,D, respectively). For the Saut d’Eau study site, a direct positive correlation was observed between estimated parasite density (by PET-PCR) and HRP2 levels, though the correlation was low (R^2^ = 0.23), and 20/26 (76.9%) of HRP2 antigen positives were not PET-PCR positive (Supplementary Figure 2).

**Figure 2:**
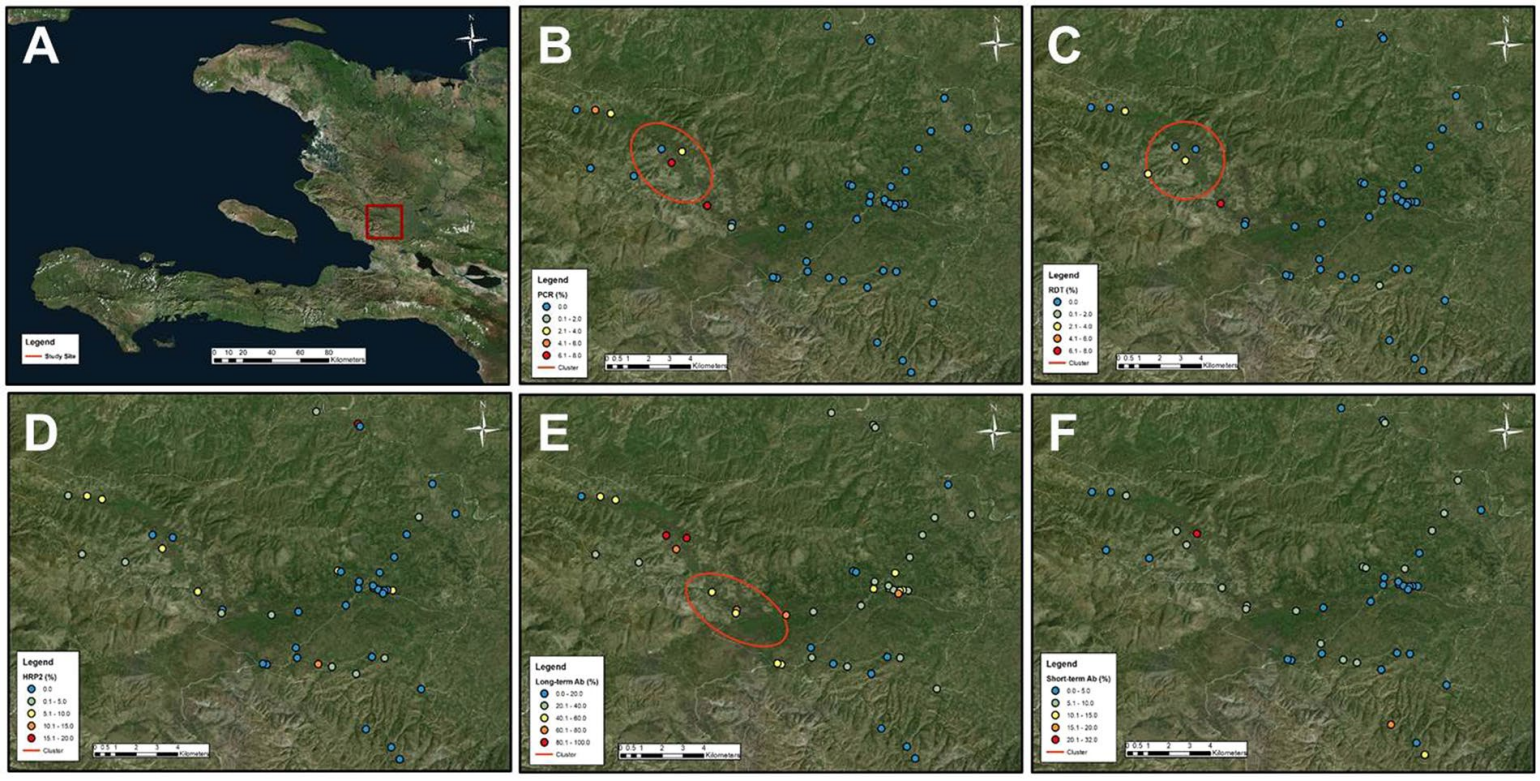
Prevalence of different measures of malaria transmission: Saut d’Eau commune, Haiti. Panels display inset area (**A**), and prevalence of: PET-PCR positives (**B**), RDT positives (**C**), HRP2 lab assay positives (**D**), long-term anti-malaria IgG positives (**E**), short-term anti-malaria IgG antibody positives (**F**). All ellipses indicate statically significant geospatial cluster of prevalence (p < 0.05). Cluster analysis was performed using the software SaTScan (Version 9.6; www.satscan.org), and SaTScan outputs were mapped on geospatial surfaces with ArcGIS v10.6 (Esri, Redlands, CA; www.esri.com).

Within each study site, heterogenous spatial patterns were observed for evidence of current or past exposure of the children to *Pf*. In Saut d’Eau, of 53 schools, 32 (60.4%) were lacking any children who were malaria test positive at the time of enrollment, and significant clustering of positives by RDT (p < 0.05) and PET-PCR (p < 0.001) was only found in the western section of the commune (Fig. 2). When compared to RDT and PET-PCR prevalence, children with short- and/or long-term IgG seropositivity were observed in a greater number of schools with 51 (96.2%) schools having at least one child who was antibody positive. However, only long-term IgG (p < 0.001) seroprevalence was found to provide significant geospatial clustering - again in the western part of the commune.

**Figure 3:**
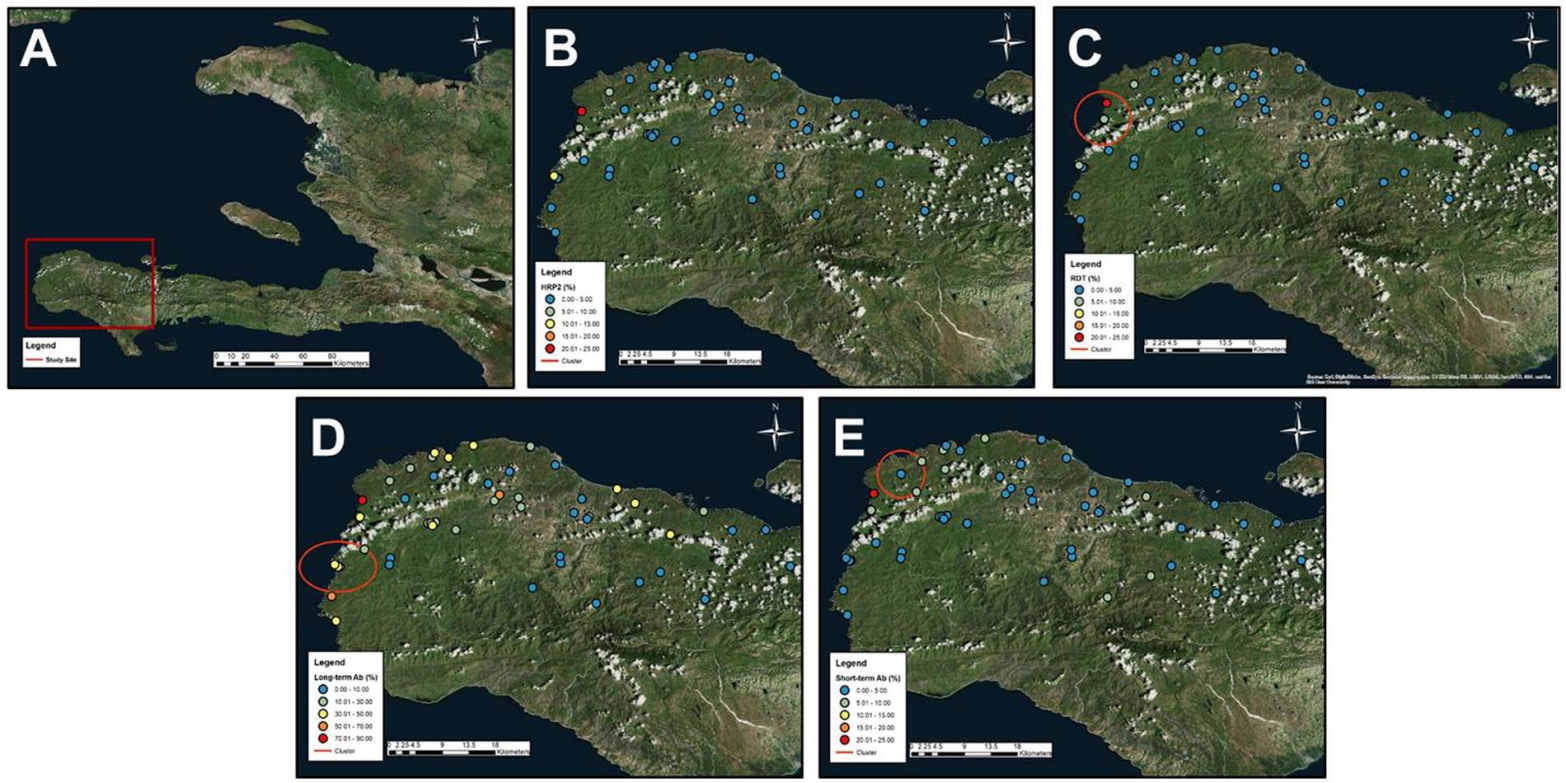
Prevalence of different measures of malaria transmission: Grand Anse department, Haiti. Panels display inset area (**A**), and prevalence of: HRP2 lab assay positives (**B**), RDT positives (**C**), long-term anti-malaria IgG positives (**D**), short-term anti-malaria IgG positives (**E**). All ellipses indicate statically significant geospatial cluster of prevalence (p < 0.05). Cluster analysis was performed using the software SaTScan (Version 9.6; www.satscan.org), and SaTScan outputs were mapped on geospatial surfaces with ArcGIS v10.6 (Esri, Redlands, CA; www.esri.com).

In Grand Anse, 17 (25.0%) of 56 schools had children who were RDT or lab HRP2 positive (Fig. 3). However, P*f* seropositivity was substantially higher with 46 (88.5%) of schools having at least one antibody positive child. Significant geospatial clustering was observed for RDT (p < 0.001), long-term IgG (p < 0.001), and short-term IgG (p < 0.001) positivity – all three along the western coast of the department.

**Figure 4:**
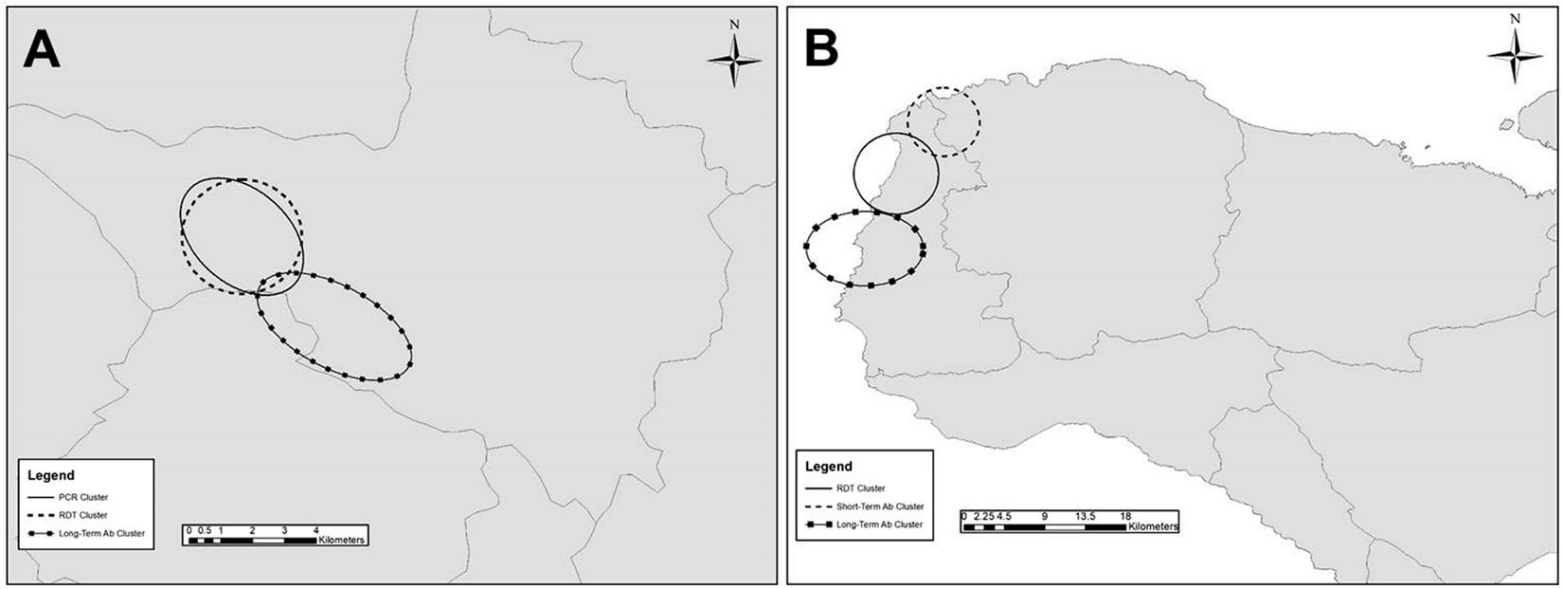
Overlay of all statistically significant clusters for each study site. Panels shown for Saut d’Eau commune (**A**) and Grand Anse department (**B**). All ellipses indicate statically significant cluster of prevalence (p < 0.05) for indicated malaria test. Cluster analysis was performed using the software SaTScan (Version 9.6; www.satscan.org), and SaTScan outputs were mapped on geospatial surfaces with ArcGIS v10.6 (Esri, Redlands, CA; www.esri.com).

When overlaying all prediction ellipses, similar areas in each study site were found to have significant clustering for different biomarkers of *Pf* infection or exposure. For the Saut d’Eau commune, the PET-PCR, RDT, and long-term IgG significant prediction ellipses were all contiguous to each other or overlapping, with the PET-PCR and RDT clusters basically superimposed on each other (Fig. 4A). In Grand Anse, the RDT, short-term IgG, and long-term IgG clusters had little overlap with each other, but all were found along the western coast of the department and contiguous (Fig. 4B).

## Discussion

It is understood that no single test or assay is able to provide a complete picture of malaria transmission or prevalence in a human population. Historical metrics for malaria include: parasite rate (as measured by number of *Pf* infections per annum / 1000 persons at risk), entomological inoculation rate (EIR, as measured by infectious bites per person per year), and parasite prevalence (typically calculated in cross-sectional fashion as percent persons infected divided by all persons in the study)^20^. These metrics have typically relied on visible identification of parasites performed by well-trained microscopists and involve a high-level of engagement and time in the field. Development and deployment of the lateral flow antigen-based malaria RDT has allowed for more pragmatic field confirmation of infection in many settings, and these tests have been reliably shown to be at least as sensitive as microscopy^10,21–23^. More recently, several laboratory assays have become more robust in identifying *Pf* biomarkers – whether these are molecules produced by the parasite during infection, or the human adaptive immune response to infection^12,17^. In both field and laboratory settings, improvement of diagnostics and assays has led to higher-throughput processing allowing a higher percentage of the sample population to be tested. The availability of multiple types of host and/or parasite biomarker data can provide better resolution in defining geospatial patterns of endemic malaria in a population and improve confidence in these data. However, it must be taken into account which data is being presented for estimates of malaria in a population as different biomarkers are measuring inherently different components of either the parasite or the host response to parasites.

Malaria elimination settings pose a unique challenge for defining endemic patterns of malaria transmission in a population. It has been observed that the last vestiges of malaria tend to reside in marginalized and isolated populations without adequate access to healthcare, and these persons may not exhibit expected treatment-seeking behavior even if healthcare opportunities are available^24–26^. With malaria elimination, it is likely that laboratory capacity for the diagnosis of malaria will be diminished, given the rarity of malarial disease^19,27^. Malaria transmission patterns in an elimination setting (and even in a control setting) are geographically heterogeneous^28,29^, and accurate estimates for these patterns are deficient if only a single indicator is utilized. In this study, five different indicators of *Pf* infection or exposure were employed in order to estimate concordance in geospatial patterns in two separate low-transmission settings (<2.0% parasite prevalence as estimated by field diagnostics).

Not surprisingly, many more Haitian children were found to be positive for IgG antibody markers of previous *Pf* exposure when compared to parasite markers of active infection (antigen or DNA). Between both study sites, 26.6% (771/2,894) of children were positive for IgG against any of the four *Pf* antigens used in the immunoassay panel, whereas only 1.9% (56/2,894) of children were antigen or DNA positive. Detection of antibodies against *Pf* antigens has been utilized by many groups to estimate transmission in endemic settings, and has the advantage of detecting malaria exposure long after parasites have been cleared from the host^30–32^. The antigens chosen for the IgG detection panel in this study included two “short-term” markers (circumsporozoite protein and liver stage antigen 1, CSP and LSA1) which would elicit an antibody response that would be predicted to last only a few months, and two “long-term” markers (merozoite surface protein 1 and apical membrane antigen 1, MSP1 and AMA1) that typically provide an IgG response lasting for years following infection^33^. Since the oldest children in this study were only seven years of age, positivity to either of the long-term markers would be a reliable proxy for lifetime *Pf* exposure. Antibody seroprevalence was found to be a useful metric for geospatial mapping, with areas of long-term IgG seropositivity found to be spatially significant for both study sites, and short-term IgG significant in Grand Anse. Since previous clinical history for these children is not known, previous *Pf* episodes can only be assumed from the IgG data, but long-term IgG seropositivity was much more prevalent when compared to short-term: 25.4% versus 3.7% - with only 1.5% of children being positive for short-term IgG alone. No previous clinical data is available for these children or populations, so it would be unknown if *Pf* occurs in outbreak cycles in the study population or is more characterized by sustained transmission.

In Saut d’Eau, both DNA and RDT data demonstrated statistically significant clustering whereas in Grand Anse only RDT results showed significant geospatial clustering. The consistency of the RDT results in identifying significant clusters is encouraging since these tests are already being used in the field for identification and appropriate treatment of *Pf* malaria, but results can also be helpful for refining epidemiological estimates. Malaria RDTs have been used for these dual purposes many times before^34–36^, and the best-in-class tests based on detection of HRP2 have been found to consistently perform well^37^. In this current study, 0.80% (23/2,894) of children were found to be RDT positive, whereas 1.7% (50/2,894) of children were positive by the HRP2 laboratory test. This finding is not surprising in a non-healthcare setting, as low amounts of circulating HRP2 antigen may result from a low-density infection, or could simply be antigen lingering in the blood from a previous *Pf* infection^15^. RDTs are meant to detect clinically-relevant parasite densities, and indeed many of these HRP2 + samples from RDT- children had very low concentrations of the antigen^38^. Among the three tests for active or very recent infection (PET-PCR, RDT, HRP2 lab assay), all confirmed the low overall prevalence of *Pf* at these sites in Haiti. However, among these indicators and the IgG measurements, very stark differences were noted for prevalence estimates within the same study site. Numerous behavioral, environmental, and population risk factors for malaria exposure could help explain these differences and future studies should capture this information for analytical purposes.

Specifically for western Saut d’Eau, the geographical uniformity of DNA, RDT, and long-term IgG prevalence points towards underlying environmental and/or human population mechanisms to allow relatively more malaria transmission in this area. Of particular note was PCR prevalence exceeding 6% and IgG seroprevalence exceeding 80% in a handful of these western schools. These findings accentuate the heterogeneous nature of *Pf* endemicity^35,39^ as schools less than 10 km from this western cluster had radically different (lower) prevalence estimates for students with active infection and historical exposure. During these school-based surveys, demographic variables were not collected, though some information can still be inferred with knowledge of school location. All schools in this western cluster were outside of urban areas, and many were located in a low-elevation valley (see Fig. 2) – both factors which are known to support the lifecycle of the *Anopheline* vector^40–42^. Even with that stated, other rural and low-elevation schools in the same commune did not show these same higher levels for malaria prevalence and exposure, emphasizing that favorable vector conditions are not a guarantee of increased malaria transmission.

These analyses are limited by the fact that this is a cross-sectional sampling design covering only a very narrow age range of the population (6–7 year olds). Malaria transmission can be very dynamic in both place and time, and given only one point in time is represented by these data, these results may not be necessarily indicative of future *Pf* incidence. Additionally, we note that history of residence or travel history was not captured for these children, so *Pf* exposure and IgG acquisition may have occurred at another location distant to the current study area. Given this concern, geospatial estimates from this study were compared to health-facility (HF) based reporting as an external validation to show where *Pf* malaria is occurring in Haiti. The spatial estimates from this study were very closely aligned with the HF reports, especially along the western coast of Grand Anse which currently reports the highest number of *Pf* cases in Haiti^19^(and unpublished data from national malaria control program). The concordance of community and HF estimates, as well as the concordance among multiple field and lab *Pf* metrics, can provide high confidence for focusing on specific geographical areas for malaria control and elimination efforts. As these DBS samples were collected years ago, any assumptions from these geospatial analyses regarding the current transmission of a dynamic pathogen like *Pf* would not necessarily hold true, and new surveys would need to be performed at this time to make contemporary estimates for these areas in Haiti.

Assessing the concordance of separate independent indicators for malaria infection and exposure can provide robust evidence for better understanding malaria epidemiology and the geospatial overlap of independent metrics provides high confidence for detecting foci of historical and ongoing transmission. For Haiti and other countries working towards malaria elimination, combining numerous data types, as demonstrated in this study, provides more refined information on malaria that can be of potential benefit to elimination programs.

## Methods

### Human subjects

Samples were collected in Haiti in May 2015 from the Saut d’Eau commune and in April 2016 from the Grand Anse department as part of a lymphatic filariasis (LF) transmission assessment survey^27^, with integration of malaria RDTs and soil-transmitted helminth microscopy of stool specimens^43^. Before enrollment, informed consent forms were sent home with each of the students so parent or legal guardian could read and sign for return to the study teams. Before sample collection, the informed consent forms were signed by the children’s parent, and verbal assent was given by the child participants. Fingerprick blood was collected on filter papers (TropBio filter wheels, Cellabs, Sydney, Australia), dried (creating a dried blood spot, DBS), and packaged individually with desiccant for later laboratory analysis at the Centers for Disease Control and Prevention in Atlanta, GA. The study protocol was approved by the National Bioethics Committee of Haiti, and this activity was considered a program evaluation activity by CDC Human subjects office (#2014–256). Persons consented to future laboratory testing of DBS, and CDC laboratory staff did not have access to any personal identifiers. All methods were carried out in accordance with relevant guidelines and regulations.

### Survey design

Surveys were conducted in evaluation units (EUs) that had met World Health Organization^44^ criteria to conduct LF TAS, with the current WHO recommendation to conduct a school-based TAS in areas where the net primary-school enrollment rate is ≥75%. Haitian school enrollment data for 2014 was utilized along with population census data to determine sampling approach employed in each EU, which are program defined. The commune of Saut d’Eau was selected as a single EU based on high baseline prevalence of LF whereas communes in the province of Grande Anse were aggregated to form a single EU based on low baseline prevalence found during initial mapping surveys^45^. Using the tables provided by WHO^44^, the target sample size and the critical cut-off threshold was determined and if the number of positive children identified fell below the critical cut-off threshold, the recommendation was made to stop mass-drug administration (MDA) for LF. Upon completion of MDA, TAS surveys were conducted to determine whether populations have reached the critical threshold of infection prevalence (<2% antigenemia), below which LF transmission is likely no longer sustainable^43^.

### RDT testing and sample collection

Approximately 200 µl of blood from each child was collected via finger prick into an Heparin-coated tube. A 5 µL aliquot of blood was used for the rapid diagnostic test (First Response Malaria Histidine-Rich Protein II (HRP2) (II3FRC30, Premier Medical Corporation, New Jersey) and results read according to manufacturer’s recommendations. Individuals with a positive RDT result received free treatment as per the national policy in Haiti. The remainder of collected blood was applied to filter paper (TropBio, Cellabs, Australia) and dried for a minimum of four hours to create DBS, packaged in individual baggies, and stored in a cool, dry place protected from light for less than 4 weeks until shipping to CDC in Atlanta where they were stored at −20 °C until processing.

### DNA extraction and photoelectron-induced electron transfer PCR

DNA was extracted from DBS by using the QIAamp DNA Mini Kit (Qiagen, Hilden, Germany) as recommended by the manufacturer. Briefly, a DBS tab (equivalent to 10 µL whole blood) was placed into a 1.5 mL tube and processed according to instructions. The DNA was eluted in 150 µL of elution buffer and stored at −20 °C until use. All Saut d’Eau samples were screened using the multiplex PET-PCR assay as previously described^46^. Briefly, the amplification of *Plasmodium* genus (forward primer: GGCCTAACATGGCTATGACG; reverse primer: FAM-aggcgcatagcgcctggCTGCCTTCCTTAGATGTGGTAGCT) was performed in a 20 µL reaction containing 2X TaqMan Environmental buffer 2.0 (Applied BioSystems, Waltham, MA) and 125 nM each of forward and reverse primers. For each sample, PET-PCR reactions were run with 2 µL of DNA template used in the PCR reaction with the following cycling parameters: initial hotstart at 95 °C for 10 min, followed by 45 cycles of denaturation at 95 °C for 10 sec, annealing at 60 °C for 40 sec. The cycle threshold (CT) values were recorded at the end of annealing step and a positive CT value was considered below 40.0. All assays were performed using a Strategene Mx3000P (La Jolla, CA). To extrapolate an estimated parasite density (in parasites per microliter blood, p/µL) from a CT value, a standard curve was made using extracted DNA from the 3D7 culture strain previously quantified by microscopy^46^.

### Blood elution from DBS and antigen detection assay

A single DBS tab (10 µL whole blood) was rehydrated in a blocking buffer (PBS pH 7.2, 0.5% Polyvinyl alcohol (SigmaAldrich, St. Louis, MO) 0.5% polyvinylpyrrolidine (SigmaAldrich), 0.1% casein (ThermoFisher, Waltham, MA), 0.5% BSA (SigmaAldrich), 0.3% Tween-20, 0.05% sodium azide, and 0.01% *E. coli* extract to prevent non-specific binding) to a final dilution of 1:20×. The presence and quantification of IgG^32^ and HRP2 antigen^38^ was performed with methodology similar to that described previously using the bead-based Luminex based MAGPIX platform (Luminex Corp., Austin, TX).

For the HRP2 detection assay, a carboxylated bead set (Bio-Plex beads, Bio-Rad, Hercules, CA) was coated by the EDC/Sulfo-NHS intermediate reaction with anti-HRP2 antibodies (Abcam mouse IgG anti-*P. falciparum* HRP2 IgG) at 20 µg per 12.5×10^6^ beads. Biotinylated detection antibodies (Abcam, a 1:1 mixture of mouse IgG anti-*P. falciparum* HRP2 and mouse IgM anti-*P. falciparum* HRP2) were prepared by Thermo Scientific EZ-Link Micro Sulfo-NHS-Biotinylation Kit (ThermoFisher) according to the manufacturer’s protocol and stored at 4 °C at a final concentration of 1 mg/mL. All reagents were diluted in buffer containing PBS, 0.05% Tween20, 0.5% Bovine Serum Albumin (SigmaAldrich), and 0.02% NaN_3_. Samples (50 µL of 1:20x dilution whole blood) were incubated with anti-HRP2 coated beads for 90 min at room temperature under gentle shaking protected from light in MultiScreen-BV filter plates (MilliporeSigma, Darmstadt, Germany). After three washes (wash buffer: PBS, 0.05% Tween 20), beads were incubated with 50 µL biotinylated detection antibody (1:500) for 45 min with same incubation conditions as above. After three washes, 50 µL streptavidin-phycoerythrin (Invitrogen, Waltham, MA) was added to all wells (1:250x of 1 mg/mL) for a 30 min incubation. After three washes, samples beads were incubated with 50 µL reagent buffer for 30 min, washed once, and resuspended in 100 µL PBS. Assay plates were shaken briefly and read on a Bio-Plex 200 machine (Bio-Rad) by generating the median fluorescence intensity (MFI) for 50 beads. The final measure, denoted as MFI-bg, was reported by subtracting MFI values from beads on each plate only exposed to sample diluent during the sample incubation step. The MFI-bg threshold for a true positive HRP2 assay signal was ascertained if the sample MFI-bg was higher than the mean + 3 SD of the MFI-bg signal of a panel of known negative DBS samples. To translate between a MFI-bg value and antigen concentration for classified positive samples, equations for a standard curve of recombinant HRP2 were calculated. Recombinant PfHRP2 antigen was provided by Microcoat Biotechnologie GmbH, and lyophilized preparations were rehydrated according to the manufacturer’s instructions.

### IgG Antibody Detection Assay

Four separate bead regions (Bio-Plex) were coupled with malaria antigens for IgG capture and subsequent detection. The antigens in the multiplex panel have all been reported on before^32,47^, and were the *Plasmodium falciparum* circumsporozoite protein (NANP)_5_ repeat (CSP, coupled at pH 5 at 30 µg/mL), liver stage antigen 1 Pl1043 epitope (LSA1, coupled at pH 5 at 60 µg/mL), merozoite surface protein 1 19kD fragment (MSP1, coupled at pH 5 at 20 µg/mL), and apical membrane antigen 1 N-terminal region (AMA1, coupled at pH 5 at 20 µg/mL).

Samples (50 µL of 1:200x dilution whole blood) were incubated with beads for 90 min at room temperature under gentle shaking protected from light in MultiScreen-BV filter plates (MilliporeSigma). After three washes (wash buffer: PBS, 0.05% Tween 20), beads were incubated with 50 µL biotinylated detection antibody (a mixture of 1:500 anti-hIgG and 1:625 anti-hIgG_4_, both produced by Southern Biotech, Birmingham, AL) for 45 min with same incubation conditions as above. After three washes, 50 µL streptavidin-phycoerythrin (Invitrogen) was added to all wells (1:250x of 1 mg/mL) for a 30 min incubation. After three washes, samples beads were incubated with 50 µL reagent buffer for 30 min, washed once, and resuspended in 100 µL PBS. Assay plates were briefly shaken and read on a Bio-Plex 200 machine (Bio-Rad) by generating the median fluorescence intensity (MFI) for 50 beads. The final measure, denoted as MFI-bg, was reported by subtracting MFI values from beads on each plate only exposed to sample diluent during the sample incubation step. The MFI-bg threshold for true positive IgG assay signal was ascertained if the sample MFI-bg was higher than the mean + 3 SD of the MFI-bg signal of a panel of known negative DBS samples.

### Geospatial analysis and mapping

Cluster analysis was performed using the software SaTScan (Version 9.6) which employs Kulldorff’s spatial scan statistic. The software compared multiple moving windows with variable radii ranging from the smallest observed distance to a pre-determined upper bound across the study area. Likelihood ratios were used to test whether there was an elevated seropositivity inside each window compared to outside the window^48^. The window with the maximum likelihood was the most probable cluster. P-values for these clusters were determined using Monte Carlo hypothesis testing. Clusters in this study were determined using circular and elliptical windows with a minimum size of two cases and a maximum size of 50% of the population, discrete purely spatial Poisson modeling, 999 Monte Carlo simulations, non-overlapping windows, and an alpha level of 0.05. SaTScan outputs were mapped on geospatial surfaces with ArcGIS v10.6 (Esri, Redlands, CA).

### Statistics

Significant differences in in IgG antibody signal by PET-PCR, RDT, or HRP2 antigen test positivity was determined by *t*-test of log-transformed assay signal values by the PROC TTEST procedure in SAS v9.4 (SAS Institute, Cary, NC). Regression comparing PET-PCR to HRP2 antigen concentration was performed in Excel.

### Disclosure

The findings and conclusions in this report are those of the authors and do not necessarily represent the official position of the CDC.

## Supplementary information

Supplementary Information.

## Data Availability

All data are available upon reasonable request.
