# Supplementary material for: Combination of Serological, Antigen Detection, and DNA Data for Plasmodium falciparum Provides Robust Geospatial Estimates for Malaria Transmission in Haiti
Source: Sci Rep. 2020 May 21;10:8443. doi: 10.1038/s41598-020-65419-w (PMC7242420; doi:10.1038/s41598-020-65419-w)
Supplement: Supplementary file 1 — Supplementary Information. [file 41598_2020_65419_MOESM1_ESM.docx]

**Combination of Serological, Antigen Detection, and DNA Data for *Plasmodium falciparum* Provides Robust Geospatial Estimates for Malaria Transmission in Haiti**

Adan Oviedo, Alaine Knipes, Caitlin Worrell, LeAnne M. Fox, Luccene Desir, Carl Fayette, Alain Javel, Franck Monestime, Kimberly Mace, Michelle A. Chang, Venkatachalam Udhayakumar, Jean F. Lemoine, Kimberly Won, Patrick J. Lammie, Eric Rogier

**Supplementary Information**


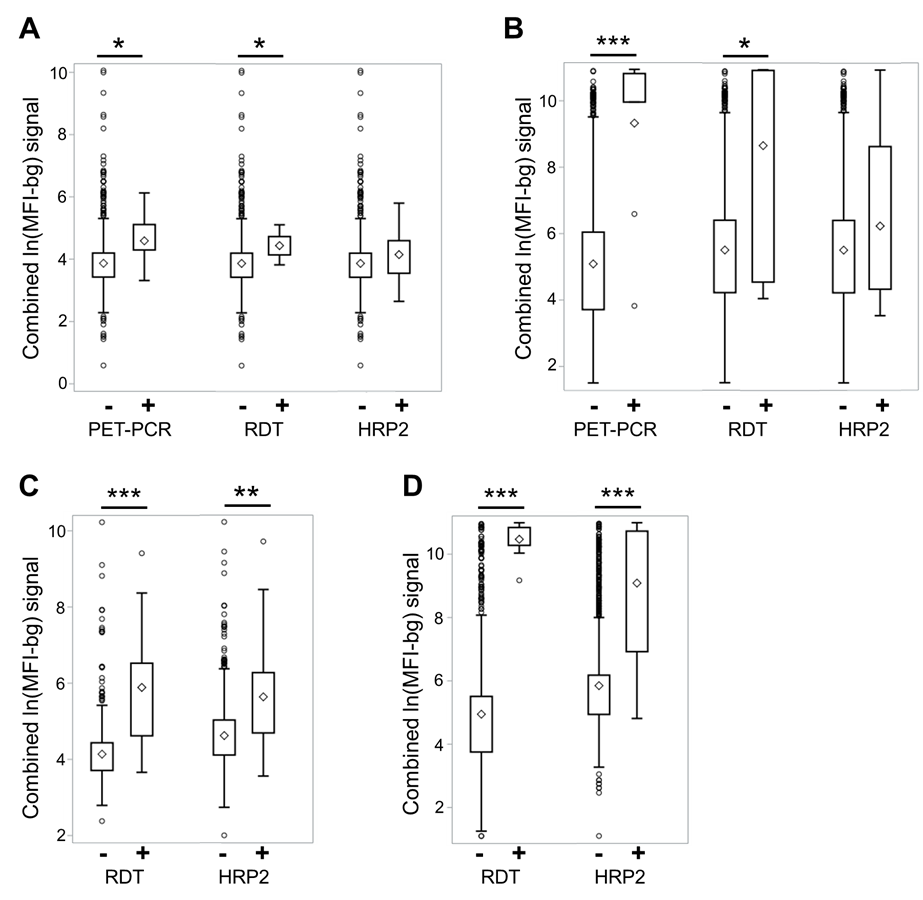


**Supplementary Figure 1. Short- and Long-Term IgG Antibody Levels by Different Test Results.** Saut d’Eau commune short-term (A) and long-term (B) anti-*Pf* antibody levels by PET-PCR, RDT, and HRP2 lab assay diagnostic test results. Grand Anse department short-term (C) and long-term (B) anti-*Pf* antibody levels by RDT and HRP2 lab assay results. Boxplots display mean as diamond and boxes extend from 25^th^ to 75^th^ percentiles. Whiskers extend +/- 1.5 interquartile range with markers outside of this range. * p < 0.05, ** p < 0.01, *** p < 0.001


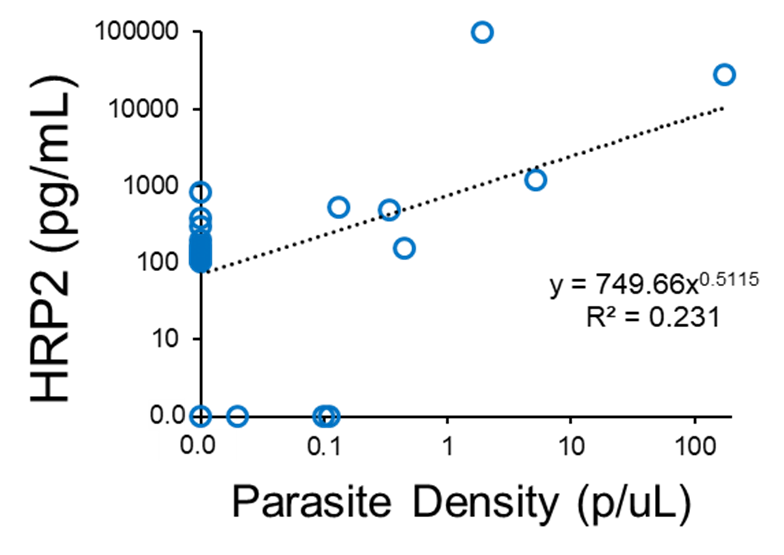


**Supplementary Figure 2. Correlation of PET-PCR Estimated Parasite Density and HRP2 Antigen Concentration.** Linear regression for the correlation between these two biological components of *P. falciparum* within the same child’s blood sample.
